# Editorial: Immunomics of the immune regulatory networks in the one health perspective

**DOI:** 10.3389/fimmu.2024.1534442

**Published:** 2025-01-07

**Authors:** Lucia Santorelli, Laxmikanth Kollipara, Marianna Caterino, Michele Costanzo

**Affiliations:** ^1^ Department of Oncology and Hematology-Oncology, University of Milan, Milan, Italy; ^2^ Leibniz-Institut für Analytische Wissenschaften (ISAS) e.V, Dortmund, Germany; ^3^ Department of Molecular Medicine and Medical Biotechnology, University of Naples Federico II, Naples, Italy; ^4^ CEINGE–Biotecnologie Avanzate Franco Salvatore, Naples, Italy

**Keywords:** immunomics, multiomic analysis, immune regulation, proteomics & bioinformatics, one health

The field of immunomics has garnered flourishing interest among the scientific community. With the advances of technological applications based on omics techniques, the immunomics field is being largely investigated to inspect the immune landscape of diseases and their intricate regulatory networks (1–4). Especially in multi-factorial contexts defined as one-health approaches, the critical link between metabolism, genome, environment, and immunity is highlighted to offer a novel perspective in which the intersection of few fields may bring the simplistic view of a disease to a multilevel dissection (5, 6). Applying diverse methodologies connected with the omics field, including genomics, proteomics and metabolomics is, in fact, a winning choice to uncover fine regulatory mechanisms of a biological system, including immune cells and mediators (1, 7, 8). In particular, these can clarify the specific interactions and networks within the immune systems of humans and animals, yielding valuable insights into health threats like zoonotic diseases, antimicrobial resistance, and food safety (5, 9). Additionally, immunomics research produce high-dimensional and high-throughput data that enable the development of computational and mathematical models, which can identify new immune targets, refine existing or novel therapies, and produce alternative, sustainable resources to enhance global health security across the human–animal–environment interface (10).

In this scenario, our recent Research Topic consists of eight articles that explore through the application of omics and bioinformatics technologies diverse facets of the immune cell landscape in many pathological conditions.

Specifically, two articles focused on autism spectrum disorder (ASD). Li et al. suggested that the immune dysregulation, particularly involving monocytes and related cells, may contribute to ASD development and be used as potential target for diagnosis and treatment. The authors analyzed six Gene Expression Omnibus (GEO) blood transcriptome datasets from ASD patients, focusing on the expression profiles of hub genes and immune cells. Then, they employed a comprehensive bioinformatic approach, which included protein-protein interactions (PPIs), functional enrichment, and other validation techniques to discover an up-regulation of monocytes and non-classical monocytes in ASD. Predictive analysis showed that three immune cell types, namely monocytes, M2 macrophages, and activated dendritic cells, had different degrees of correlation with 15 identified hub genes. Further, miRNA-mRNA network and agent-gene interaction analyses identified two miRNAs (miR-342-3p and miR-1321) and 23 agents connected with ASD.

In a complementary approach by Ma et al., ASD-relevant datasets retrieved from GEO database and immune-relevant genes were analyzed by a comprehensive bioinformatics approach to find correlation between immune cells and ASD. The authors established an accurate correlation between neutrophils and ASD, identifying 14 pleiotropic regions which contained six potential candidate genes. The authors detailed the CFLAR gene having a specific expression pattern in neutrophils, suggesting it as a biomarker for ASD pathogenesis.

Immunomics can provide effective findings to address threats of human health such as infectious and zoonotic diseases, or antimicrobial resistance. Expanding the connection between immune networks and infectious agents, Pereiro et al. found by transcriptomic and metatranscriptomic profiling that zebrafish exposed to sulfamethoxazole (SMX) and clarithromycin (CLA) at environmental concentrations had higher bacterial load in the intestine and kidney. In particular, the impact on the intestine was more pronounced than kidney, revealing that a major immune dysregulation affects the complement/coagulation system. In addition, SMX+CLA-treated fishes were not able to activate the transcription of complement-related genes when infected with spring viremia of carp virus. These findings suggested that antibiotics at environmental concentrations increase the susceptibility to virus infection, with the impairment of the complement system playing a pivotal role in antibiotic-mediated immunosuppression.

Chen et al. addressed the clinical challenge of identifying predictive biomarkers for the efficacy of antiviral treatments in patients with chronic hepatitis B virus (HBV) infection, through untargeted metabolomics in plasma. The study identified several metabolites with high diagnostic potential to distinguish high-responder from low-responder patients. In particular, four metabolites, namely 2-methyl-3-ketovaleric acid, 2-ketohexanoic acid, 6-oxo-1,4,5,6-tetrahydronicotinic acid, and α-ketoisovaleric acid showed very high sensitivity and specificity. Globally, the authors observed variation in key metabolites with the potential to support anti-HBV innate immunity or anti-inflammatory effects, providing new perspectives for treating and preventing inflammatory diseases.

Rankawat et al. revealed a defined circadian regulation within host response pathways and immunological networks that influences the progression and severity of clinical symptoms in malaria. Through the systematical analysis of rhythmic patterns in serum, plasma, and tissue proteins dysregulated in malaria, the authors mapped the rhythmicity of host proteins and immune factors involved in malaria pathogenesis. Their findings demonstrate that several components within the host physiological pathways and immunological networks activated in malaria show 24-hour rhythms across species, with rhythmic immunity genes showing significant overlap among mice, non-human primates, and humans. This cyclic regulation shows importance in the management of host-parasite interactions and to establish possible defense mechanisms in malaria.

In another Research article, Li et al. investigated the protective effects of chitosan oligosaccharides (COS) in a mouse model of premature ovarian failure (POF) induced by cyclophosphamide and busulfan. Findings suggest that COS supports ovarian function by reducing oxidative stress, regulating immune responses, and improving reproductive hormone levels, which collectively help protect against POF. The study highlights the potential of COS as a therapeutic agent for preserving ovarian health and mitigating reproductive aging effects in POF.

An exhaustive review by Gao et al. reported how immunomics can explain the complex immunological interactions between humans, animals, and environments in the one-health perspective with application to the recent COVID-19 pandemic. In fact, significant technological advancements in immune profiling and in the knowledge of immune pathways have enhanced vaccine development and viral mutation tracking, expanding the understanding of zoonotic transmissions. Novel insights on the immune response provide essential knowledge about viral spread and host susceptibility mechanisms. Critical is the role of animals within their ecological niche in shaping viral epidemiology, given that environmental factors influence immune response across species, affecting viral persistence and the risks of spillover.

Another review article by Monda et al. explored the potential of ketogenic diet in reducing neuroinflammation and modulating immune responses. This work highlights how the low-carbohydrate, but high-fat and -protein diet composition is able to modify metabolic processes, dampening inflammatory pathways and supporting immune regulation through the production of ketone bodies. In this context, neurodegenerative diseases share the hallmark of chronic neuroinflammation due to the activation of the immune response in the central nervous system with proliferation of immune cells like microglia and astrocytes and release of inflammatory mediators. Actually, the authors report evidence on the beneficial role of ketogenic diet on neurodegenerative diseases based on the metabolic shift occurring from the use of glucose to ketone bodies as the principal source of energy for brain. Ketone bodies may have the potential to reduce the demyelination of nerve fibers, contribute to the restoration of the respiratory chain, improve mitochondrial function, increase the number of neuroprotective mediators, and promote brain autophagy to eliminate protein aggregates or damaged organelles. By shifting energy source, the ketogenic diet might offer a therapeutic approach for immune and brain health.

Overall, our greatest reward has been to receive such relevant contributions in this Research Topic to emphasize the importance and the high potential of immunomics strategies. Such advancements are instrumental to improve the knowledge of several aspects in immunology, including immunometabolism, immune system dynamics and regulation networks, and understand the cross-talk between immune system and human health.
